# *Escherichia coli* O157:H7 in Retail Lettuce (*Lactuca sativa*) in Addis Ababa City: Magnitude of Contamination and Antimicrobial Susceptibility Pattern

**DOI:** 10.3389/fmicb.2021.694506

**Published:** 2021-07-16

**Authors:** Aklilu Feleke Haile, Silvia Alonso, Nega Berhe, Tizeta Bekele Atoma, Prosper N. Boyaka, Delia Grace

**Affiliations:** ^1^Aklilu Lemma Institute of Pathobiology, Addis Ababa University, Addis Ababa, Ethiopia; ^2^Department of Veterinary Biosciences, The Ohio State University, Columbus, OH, United States; ^3^International Livestock Research Institute (ILRI), Addis Ababa, Ethiopia; ^4^Ethiopian Public Health Institute, Addis Ababa, Ethiopia; ^5^Department of Veterinary Biosciences, College of Veterinary Medicine, The Ohio State University, Columbus, OH, United States; ^6^Department of Microbial Immunity and Infection, The Ohio State University, Columbus, OH, United States; ^7^Infection Diseases Institute, The Ohio State University, Columbus, OH, United States; ^8^International Livestock Research Institute (ILRI), Nairobi, Kenya; ^9^Natural Resources Institute, Chatham, United Kingdom

**Keywords:** Addis Ababa, antimicrobial, *Escherichia coli* O157:H7, lettuce, prevalence

## Abstract

*Escherichia coli* O157:H7 is an important foodborne pathogen but largely under investigated in Africa. The objectives of this study were to estimate the prevalence and pattern of antimicrobial resistance of *E. coli* O157:H7 in lettuce in Addis Ababa, Ethiopia. A total of 390 retail lettuce samples were collected across the 10 subcities of Addis Ababa. *E. coli* O157:H7 was isolated and identified following ISO-16654:2001 standard. The isolates were further tested for antimicrobial susceptibility to 13 antimicrobials using the Kirby–Bauer disk diffusion method. Out of the 390 lettuce samples examined, two (0.51%) carried *E. coli* O157:H7. The antimicrobial susceptibility pattern of strains showed resistance to ampicillin (100%) and tetracycline (50.0%). One of the two isolates was multidrug resistant to two antimicrobials tested. The results of this study demonstrate the presence of drug-resistant *E. coli* O157:H7 in lettuce in markets in Addis Ababa. Despite the low prevalence, its presence in a product that is eaten raw highlights potential public health risk in the area associated with this pathogen.

## Introduction

Foodborne illnesses are a crucial cause of morbidity and mortality, and a big impediment to socioeconomic development worldwide (WHO, 2015). The incidence of foodborne disease appears to be increasing globally (Jaffee et al., 2018). For industrialized countries, it has been estimated that up to one-third of the population suffers at least one foodborne episode each year. Considering the problems of sanitation in the developing world, the prevalence of foodborne disease can be assumed to be even higher (Käferstein, 2003; Grace, 2015).

*Escherichia coli* is a bacterium that colonizes the intestinal track of animals and humans. Most strains of *E. coli* are harmless and are considered part of the normal gut flora. However, specific strains such as *E. coli* O157:H7 have the ability to produce toxins that can cause severe illness (Wang et al., 2014). *E. coli* O157:H7 infection in humans has different clinical manifestations ranging from asymptomatic (carrier state) to more complicated forms of the disease that includes hemorrhagic colitis (HC), hemolytic uremic syndrome (HUS), thrombotic thrombocytopenic purpura (TTP), and even death. While the disease can affect any age group, its most severe forms are mostly seen in children less than 5 years, elders greater than 65 years, and immunocompromised individuals (Boyce et al., 1995; Mead and Griffin, 1998; Moses et al., 2006).

Consumption of vegetables contaminated with *E. coli* O157:H7 has been associated with many outbreaks around the world. Lettuce has been associated to many outbreaks of *E. coli* O157:H7. In the United States, outbreaks of *E. coli* O157:H7 infections have been caused by lettuce and alfalfa sprouts (Laven, 2006). Lettuce has been associated to many outbreaks of *E. coli* O157:H7. Contamination of lettuce with foodborne pathogenic microorganisms can occur at several points along the value chain, from the field to the market (Barak et al., 2002; Lelieveld et al., 2003). The use of manure from cattle as fertilizer or the close proximity of the vegetable fields or water supplies to cattle farms have been associated with contamination of vegetables (Elder et al., 2000; Ibenyassine et al., 2007).

Antibiotic resistance is an increasingly concerning health issue across the globe (Käferstein, 2003). Cattle and other ruminants can be asymptomatic carriers of *E. coli* O157:H7 (Meng and Doyle, 1998) and, through exposure to antimicrobial drugs in the livestock production system, may serve as a source of antimicrobial-resistant bacteria.

The three major types of vegetable retailers in Addis Ababa are roadside retailers (individuals selling in improvised spots or basic stalls along the streets), open market retailers (sellers with basic stalls within an open market), street shops (within a fixed premise along the street), and supermarkets. Addis Ababa has two large open markets that serve as central points for the distribution of vegetables coming from rural and peri-urban areas to be sold and consumed in the city. These main vegetable markets are called “Piazza” and “Mercato,” and approximately 50% of their supplies originate from smallholder producers or farmers’ cooperatives. Many other types of vegetable retailers across the city source their vegetables at these markets (Wiersinga and de Jager, 2009; Setegn, 2015).

The increasing popularity of fast food, in particular burgers, which use raw lettuce as an ingredient, as well as the use of lettuce in salad as a common side dish in Addis Ababa’s restaurants raises questions around the safety of this product and its potential health risks. No information is available on the magnitude of contamination and antimicrobial susceptibility patterns of *E. coli* O157:H7 in Addis Ababa, and in the rest of Ethiopia.

The present study was designed to estimate the prevalence of *E. coli* O157:H7 serotype in lettuce from selected lettuce retail outlets in Addis Ababa, Ethiopia, as well as to determine the antimicrobial susceptibility pattern of *E. coli* O157:H7 serotypes.

## Materials and Methods

### Study Area

The study was carried out in Addis Ababa, the capital city of Ethiopia. The city covers 540 km^2^ and is divided into 10 subcities. The city lies at an elevation of 2,355 m above sea level and is located at 9°1′48″N 38°44′24″E. The city has a minimum, maximum, and average temperature of 14, 21, and 17.5°C, respectively. Addis Ababa has bimodal rainfall with an annual average of 1,800 mm with the highest of rain falls during the long rainy season from June to September. The short rainy season is from February to April. The capital city has an estimated human population of 3.15 million (CSA, 2010).

### Study Design and Sample Size Determination

A cross-sectional study was conducted from October 2018 to December 2019 to determine the prevalence and antimicrobial susceptibility of *E. coli* O157:H7 serotype in retail raw lettuce samples obtained from selected vegetable retail outlets, in Addis Ababa.

The sample size required was calculated according to the formula in Thrusfield (2018):

$$\text{n}={\text{Z}}^{2}{\text{P}}_{\text{exp}}\left(1-{\text{P}}_{\text{exp}}\right)/{\text{d}}^{2}$$

where Z = z statistic for level of confidence; n = required sample size; P_*e*__*x*__*p*_, the expected prevalence and; d the desired absolute precision. We used an expected *E. coli* O157:H7 prevalence of 50, 5% precision, and level of confidence of 95%. The calculated sample size required was 384.

### Sampling Methods

Addis Ababa has 10 subcities. Across the 10 subcities, 390 vegetable retail outlets were conveniently included in the study. A lettuce sample was purchased from each of the outlets in the same fashion as they are sold to the buyers.

Each lettuce sample was placed in a sterile individual plastic bag. The sample was identified by its exclusive sample identification number, which was written on the plastic bag, alongside the subcity and date of sampling. Finally, the sample was transported to the Microbiology Laboratory of the Aklilu Lemma Institute of Pathobiology, Addis Ababa University, at cold temperature in a cool box. Upon arrival to the laboratory, the samples were stored in a refrigerator at ±4°C. The samples were processed within 6–12 h from arrival. Detection of *E. coli* O157:H7 was done using an ISO-16654:2001 standard (ISO, 2001).

### Sample Preparation and Enrichment

The inner and outer leaves of the lettuce were cut by a sterile scalpel to form a 25-g sample and put in a sterile Stomacher bag. Then, 225 ml of modified Tryptone Soya Broth (TSB) supplemented with Novobiocin (mTSB + N) (1:9) was added to the lettuce and homogenized using a homogenizer (Stomacher 400, Seward Medical, England) at high speed for 2 min. The enrichment sample was then incubated aerobically at 41.5°C for 24 h.

### Isolation

Detection of *E. coli* O157:H7 was done using ISO-16654:2001 standard (Manyi-Loh et al., 2018). All enriched broths were plated onto cefixime tellurite sorbitol MacConkey agar (CT-SMAC) that was sorbitol MacConkey agar (Oxoid, England), supplemented with 0.05 mg/L cefixime and potassium 2.5 mg/L tellurite (Oxoid, England) (CT-SMAC) (Oxoid, England) and incubated at 37°C for 24 h. Following the end of the incubation period, the CT-SMAC agar plates were examined for the presence of non-sorbitol fermenter colorless colonies, and subsequently they were subcultured on Rainbow agar O157 (Hayward, United States). The plates were then incubated for 20–4 h, at 37°C and observed for the presence of typical black or gray coloration on Rainbow agar O157, which shows pure colonies (Biolog, 2008).

### Biochemical Confirmation

Five typical colonies from each Rainbow agar O157 plate were subcultured on nutrient agar (Oxoid, England) for biochemical confirmation by indole formation. The agar plates were incubated at 37°C for 18–24 h. One colony from the pure culture on the nutrient agar was inoculated into a tube of tryptone/tryptophan medium (Oxoid, England) and incubated at 37°C for 24 h. Then, 1 ml of Kovack’s (Oxoid, England) was added, and the tube was allowed to stand at room temperature for 10 min. The formation of red color indicates a positive reaction (Manyi-Loh et al., 2018).

### Serological Identification of O157 and H7 Antigens

Latex agglutination was performed for confirmation of *E. coli* O157:H7 using an *RIM E. coli* O157:H7 latex test kit (Oxoid, England) (Manyi-Loh et al., 2018). The latex kit has four compartments: latex test reagent, latex control reagent, the positive controls, and negative controls. The particle in each reagent was coated with a different antibody against *E. coli* serotype O157 or *E. coli* serotype H7, and therefore the control was coated with normal rabbit globulin. The positive and negative controls are suspensions of formalin-killed *E. coli* O157:H7 cells and non-specific *E. coli* cells, respectively.

Indole-positive colonies were examined for their serological reaction with antiserum to *E. coli* O157:H7 using an *RIM E. coli* O157:H7 latex test (Oxoid, England). Indole-positive colonies were subcultured from the nutrient agar to the sorbitol MacConkey agar (Oxoid, England). For every isolate to be tested, one drop of test latex was dispensed into a well of the test slide. In like manner, one drop of *E. coli* control latex was dispensed into a separate well of the test slide. Using a plastic stick, a portion of the non-sorbitol fermenting colony (NSFC) was removed from the sorbitol MacConkey agar-SMAC (Oxoid, England) plate, emulsified in *E. coli* O157 test latex on the slide, and spread over the reaction area. Using a fresh plastic stick, the process was repeated with the remaining NSFC and emulsified in *E. coli* control, latex on the slide. The slide was rotated using circular motions for up to 1 min or until agglutination appeared. For *E. coli* O157 positives that agglutination occurs with the *E. coli* O157 test latex and the control latex is negative, the isolate was streaked from sorbitol MacConkey agar (Oxoid, England) to a blood agar (Oxoid, England) plate and incubated at 37°C for 18–24 h. After 18–24 h of incubation, the sweep of growth from the blood agar plate was emulsified in a drop of *E. coli* H7 test latex. Colonies giving an agglutination reaction were confirmed as *E. coil* O157:H7 positive.

### Molecular Conformation of *E. coli* O157

Isolates were confirmed by polymerase chain reaction (PCR) for the O157 lipopolysaccharide O-antigen synthesis *rfbE*O157 gene. The reactions were performed using oligonucleotide primer set specific for *rfbE*O157 gene (Thermo Fisher Scientific, United States). Primers for the amplification of the *rfbE*O157 genes are *rfbE*O157-F 5′-AAG ATT GCG CTG AAG CCT TTG-3′and *rfbE*O157 R-5′-CAT TGG CAT CGT GTG GAC AG-3′ *rfbE*O157 producing 497 bp of *rfbE*O157 gene (Desmarchelier et al., 1998).

The PCR was performed using Illustra^TM^ PuReTaq^TM^ Ready-To-Go^TM^ PCR Beads (GE Healthcare Bio-Sciences, United States) in 25 μl reaction using SimpliAmp Thermal Cycler (Applied Biosystems, Singapore). The PCR reaction mixtures were subjected to the following conditions in SimpliAmp Thermal Cycler: an initial denaturation of one cycle at 95°C for 5 min, followed by 35 cycles, each consisting of 94°C for 30 s, 66°C for 30 s, 72°C for 30 s, and a final extension at 72°C for 10 min (Desmarchelier et al., 1998).

Amplification products were resolved by electrophoresis in 2% agarose gels, stained with ethidium bromide. The gel was run for at 100 V for 40 min and then visualized with UV light using AlphaImager instrument and FluorChem HD2 software. Product sizes were determined by comparison with a 100-bp gene ruler DNA ladder (Thermo Fisher Scientific, United States).

### Antimicrobial Susceptibility Testing

The antimicrobial susceptibility was performed following the standard agar disk diffusion method consistent with CLSI (2014) using commercial antimicrobial disks (Table 1).

**TABLE 1:** Antibiotic disks used to test *E. coli* O157:H7 and their respective concentrations.

No.	Antibiotic disks	Disk code	Concentration	Diameter of zone of inhibition in millimeter (mm)		
				Resistant ≤	Intermediate	Susceptible ≥
1	Ampicillin	AM	10 μg	13	14–16	17
2	Amoxycillin-clavulanic acid	AMC	20/10 μg	13	14–17	18
3	Amikacin	AK	30 μg	14	15–16	17
4	Ciprofloxacin	CIP	5 μg	15	16–20	21
5	Ceftriaxone	CRO	30 μg	19	20–22	23
6	Cefoxitin	FOX	30 μg	14	15–17	18
7	Nitrofurantoin	F/M	50 μg	14	15–16	17
8	Kanamycin	K	30 μg	13	14–17	18
9	Nalidixic acid	NA	30 μg	13	14–18	19
10	Sulfamethoxazole-trimethoprim	SXT	25 μg	10	11–15	16
11	Tetracycline	TE	30 μg	11	12–14	15
12	Streptomycin	S	10 μg	11	12–14	15
13	Gentamicin	GM	10 μg	12	13–14	15

Each isolated bacterial colony from pure fresh culture was transferred into a tube of 5 ml TSB (Oxid, England) and incubated at 37°C for 6 h. The turbidity of the culture broth was adjusted using sterile saline solution or added more colonies to get turbidity, usually comparable with that of 0.5 McFarland standards (approximately 3 × 10^8^ CFU per ml). Mueller–Hinton agar (Oxiod, England) plates were prepared consistent with the manufacturer. A sterile cotton swab was immersed into the suspension and rotated against the side of the tube to get rid of the sulplus fluid and then swabbed in three directions uniformly on the surface of Mueller–Hinton agar plates. After the plates dried, antibiotic disks were sited on the inoculated plates using sterile forceps. The antibiotic disks were gently pressed onto the agar to ensure firm contact with the agar surface, and incubated at 37°C for 24 h. Following this the diameter of the inhibition zone formed around each disk was measured using a black surface, reflected light, and transparent ruler by lying it over the plates. The isolates were classified as sensitive, intermediate, and resistant according to the breakpoints of the clinical interpretative criteria recommended by CLSI guidelines (CLSI, 2014). *E. coli* (ATCC 25922) was used as a positive control.

### Ethical Consideration

The study protocol was ethically approved by the Institutional Review Board of Aklilu Lemma Institute of Pathobiology, Addis Ababa University (Minutes Ref NO: ALIPB IRB/006/2011/2018).

### Data Management and Analysis

The data were entered and coded in MS Excel and then analyzed using IBM SPSS version 25.0 (SPSS, 2017). The prevalence was determined by dividing the number of positive samples by the total number of samples examined. Descriptive statistics such as frequency and percentages were used to describe the proportion of resistant, intermediate, or susceptible strains. The difference in prevalence by subcity was determined using chi-square (χ^2^) test. A *p*-value < 0.05 was considered indicative of a statistically significant difference.

## Results

### Prevalence

A total of 390 retail lettuces samples were collected from vegetable retailer outlets in Addis Ababa, 39 in each of the 10 subcities. Out of 390 lettuce samples examined, two (0.51%) (95% CI = −0.2–1.23) were positive to *E. coli* O157:H7.

The positive samples were from outlets in Bole and Yeka subcities. However, the prevalence of *E. coli* O157:H7 was not statistically significant (χ2 = 8.041; *df* = 9, *p* = 0.530 (*p* > 0.05) among the subcities of vegetable retail outlets (Table 2).

**TABLE 2:** Prevalence of *E. coli* O157:H7 by risk factor.

Risk factor		Number examined	Positive no (%)	χ2	*df*	*p*-Value
Sub-city	Addis Ketema	39	0	8.041	9	0.530
	Akaki Kality	39	0			
	Arada	39	0			
	Bole	39	1 (6.25)			
	Gullele	39	0			
	Kirkos	39	0			
	Kolfe Keraneo	39	0			
	Lideta	39	0			
	Nefassilk	39	0			
	Yeka	39	1 (6.25)			

### Antimicrobial Susceptibility Pattern of *E. coli* O157:H7

Both isolates were resistant to ampicillin. One of the isolates, the one from Bole subcity, showed additional resistance to tetracycline. Both isolates were susceptible to five antibiotics: amoxicillin-clavulanic acid, ceftriaxone, cefoxitin, nalidixic acid, and sulfamethoxazole-trimethoprim (Table 3).

**TABLE 3:** Antimicrobial susceptibility pattern of *E. coli* O157:H7 isolates (*n* = 2).

Antimicrobial use	Sensitive no. (%)	Intermediate no. (%)	Resistant no. (%)
Ampicillin (AM)	0 (0)	0 (0)	2 (100)
Amoxicillin-clavulanate (AMC)	2 (100)	0 (0)	0 (0)
Amikacin (AK)	1 (50)	1 (50)	0 (0)
Ciprofloxacin (CIP)	1 (50)	1 (50)	0 (0)
Ceftriaxone (CRO)	2 (100)	0 (0)	0 (0)
Cefoxitin (FOX)	2 (100)	0 (0)	0 (0)
Nitrofurantoin (F/M)	1 (50)	1 (50)	0 (0)
Kanamycin (K)	1 (50)	1 (50)	0 (0)
Nalidixic acid (NA)	2 (100)	0 (0)	0 (0)
Sulfamethoxazole-trimethoprim (SXT)	2 (100)	0 (0)	0 (0)
Tetracycline (TE)	1 (50)	0 (0)	1 (50)
Streptomycin (S)	1 (50)	1 (50)	0 (0)
Gentamicin (GM)	1 (50)	1 (50)	0 (0)

## Discussion

Our study is the first to report the presence of *E. coli* O157:H7 in lettuce in Ethiopia. The prevalence was low 0.51% (95% CI = −0.2–1.23). Studies in other countries have reported similar low prevalence. Studies have reported zero prevalence 7 in Spain and Mexico (Canizalez-Roman et al., 2013; Castro-Ibáñez et al., 2015). Higher prevalence has been reported in Pakistan (2.0%) (Shah et al., 2015) and in Iran (10.0%) (Shakerian et al., 2016).

The prevalence of *E. coli* O157:H7 was not statistically significant (*p* > 0.05) among the subcities of vegetable retail outlets. This might be due to outlets across the city source their vegetables from the same main vegetable markets, Piazza, and Mercato (Wiersinga and de Jager, 2009). The small number of positives also means a much larger sample size would be needed to identify any differences.

Antimicrobial resistance is a one health issue, with connecting the health of people, animals, and the environment in a complex web of causation (Käferstein, 2003). The *E. coli* O157:H7 isolates in this study showed resistance to ampicillin (100%) and tetracycline (50.0%). There are no other similar studies in Ethiopia. However, resistance to the same antibiotics has been reported in studies in Iran [ampicillin (100%) and tetracycline (87%)] (Shakerian et al., 2016) and in Pakistan [ampicillin (87%) and tetracycline (92%)] (Shah et al., 2015). On the other hand, among the two *E. coli* O157:H7 isolates from lettuce, one isolate showed multidrug resistance to ampicillin and tetracycline. Comparable to the present study, multidrug resistance was recorded from Iran (Shah et al., 2015; Shakerian et al., 2016). However, no studies have been found in Ethiopia. The resistance pattern might be related the broad use of tetracycline among livestock (Gemeda et al., 2020) and ampicillin in management of various infections in Ethiopia (Eguale et al., 2015).

The present study had a limitation in laboratory facility. The use of immune-magnetic separation (IMS) with enrichment in broth culture is commonly used to enhance the isolation of *E. coli* O157 from samples with an expected low concentration of bacteria (Chapman et al., 1994). In our study, we used enrichment without IMS for the isolation of *E coli* O157:H7. More general proxies of fecal contamination such as coliforms and Enterobacteriaceae would have been useful indicators of the microbiological safety of the lettuce, but this was outside the reach of our study. Nevertheless, the present study is the first to report the existence of *E. coli* O157:H7 in lettuce samples in Ethiopia and its antimicrobial resistant patterns. This information calls for larger and duly powered studies to understand the sources, the points of contamination, and define appropriate risk mitigation strategies for the country.

## Data Availability Statement

The raw data supporting the conclusions of this article will be made available by the authors, without undue reservation.

## Author Contributions

AH conceived and designed the study, conducted the study, analyzed the data, and wrote the manuscript. SA conceived the study, provided guidance to its design, and reviewed the manuscript. NB designed the study, analyzed the data, and wrote the manuscript. TB conducted the study and analyzed the data. PB reviewed the manuscript and wrote the manuscript. DG conceived and designed study, analyzed the data, and reviewed the manuscript. All authors contributed to the article and approved the submitted version.

## Conflict of Interest

The authors declare that the research was conducted in the absence of any commercial or financial relationships that could be construed as a potential conflict of interest.
